# A rare lymphoplasmacyte-rich meningioma involving the dura of the skull base and cervical spinal cord: A case report

**DOI:** 10.1515/tnsci-2022-0263

**Published:** 2022-12-13

**Authors:** Siyao Zhu, Yuting Zou, Ya Wang, Gongxun Xie, Shengying Xiao, Furen Zeng, Yichen Lu

**Affiliations:** Department of Oncology, Hunan Provincial People’s Hospital (The First Affiliated Hospital of Hunan Normal University), 89 Guhan Road, Furong, Changsha 410002, PR China; Department of Medical Oncology, Lung Cancer and Gastrointestinal Unit, Hunan Cancer Hospital/The Affiliated Cancer Hospital of Xiangya School of Medicine, Central South University, Changsha 410013, PR China; Department of Pathology, Hunan Provincial People’s Hospital (The First Affiliated Hospital of Hunan Normal University), Changsha 410002, PR China

**Keywords:** lymphoplasmacyte-rich meningioma, diffuse growth, magnetic resonance imaging, differential diagnosis, treatment

## Abstract

Lymphoplasmacyte-rich meningioma (LPRM) is a rare subtype of meningioma, the specific pathogenesis of which remains unclear. Herein, we report the case of a 48-year-old Asian man who experienced progressive deafness and limb weakness. Magnetic resonance imaging revealed extramedullary masses diffusely growing, wrapping, and compressing the cervical spinal cord. The dural lesion was partially excised by surgery, and postoperative pathological examination confirmed the diagnosis of LPRM. Diffuse LPRM is extremely rare, and its treatment is challenging owing to difficulties associated with surgery and the uncertain efficacy of traditional therapies. Therefore, further clinical practice and basic research are needed to improve the prognosis of diffuse LPRM.

## 1 Introduction

Meningiomas, which are predominantly slow-growing, are the most common primary tumors of the central nervous system [1]. These tumors can originate from the dura at any site, but most commonly arise from the skull vault, skull base, and dural reflections [2]. According to the 2016 World Health Organization (WHO) guidelines, the classification of meningiomas is based on their histological appearance, and they are divided into 15 subtypes with three grades for each subtype. Grade I meningioma is the most commonly seen in clinical practice [3,4]. Lymphoplasmacyte-rich meningioma (LPRM) is a rare histological subtype of benign (WHO grade I) meningiomas, characterized by extensive inflammatory cell infiltration and a variable proportion of meningeal epithelial neoplasms [5]. Here, we present a rare case of LPRM with diffuse lesions involving the saddle area, tentorium cerebella, and extending along the meninges of the skull base from the clivus, descending to C7 nerve root.

## 2 Case presentation

**Figure 1:**
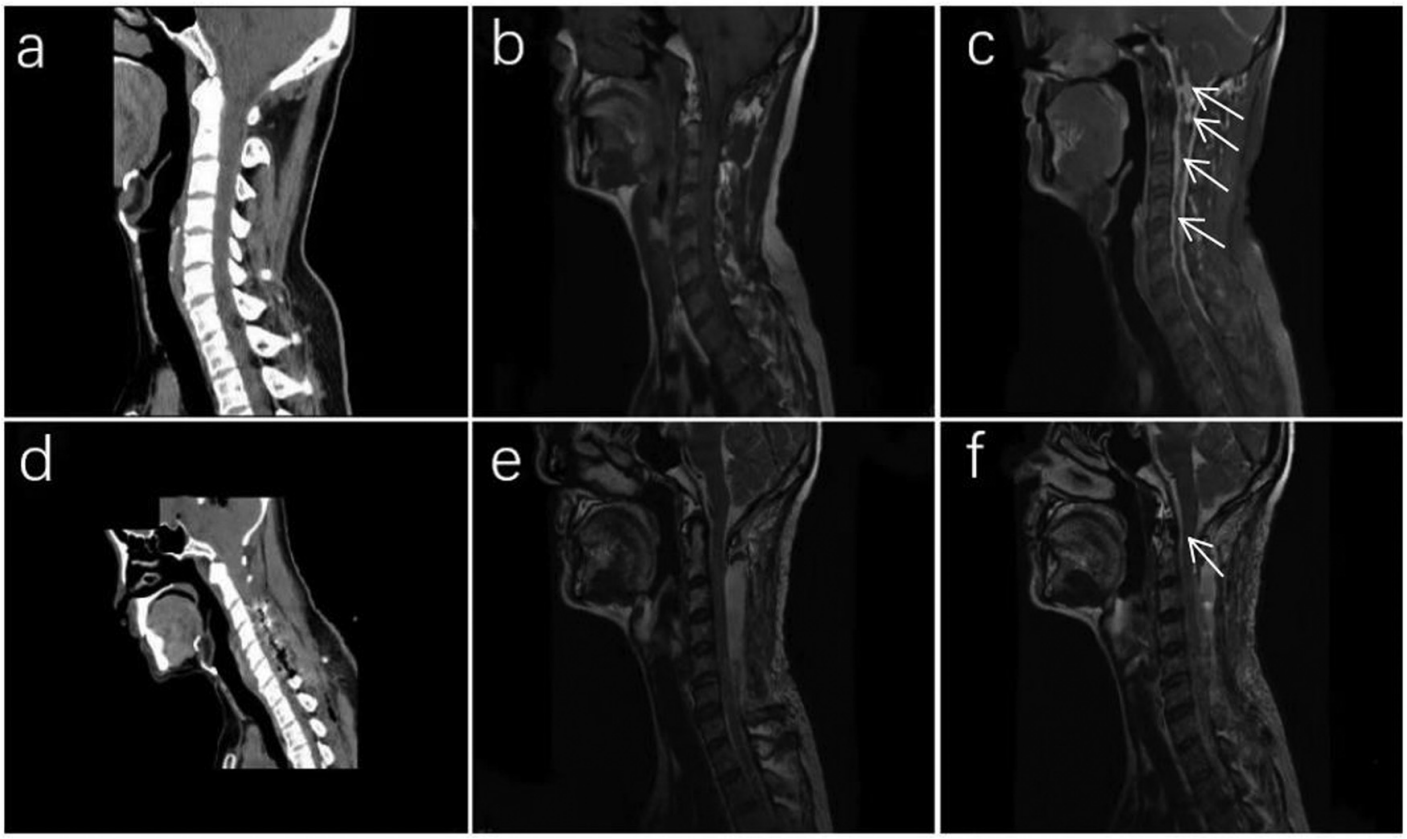
(a–c) Preoperative images of the patient: CT of the head and cervical spine showing no obvious abnormalities (a), MRI showing that the lesion extends from the tentorium cerebella to the seventh cervical spinal canal, enveloping the cervical spinal cord. The lesion is isointense on T1-weighted images and slightly longer range on T2-weighted images, enhanced remarkably and homogeneously after infusion of contrast administration (arrows) (b and c). (d–f) Postoperative images, CT showing changes in the dural lesions after resection (d). Postoperative MRI showing that a part of the thickened dura of the foramen magnum and C1 vertebral body is nodular, while nodular signals in the other regions are significantly reduced (arrows) (e and f).

**Figure 2:**
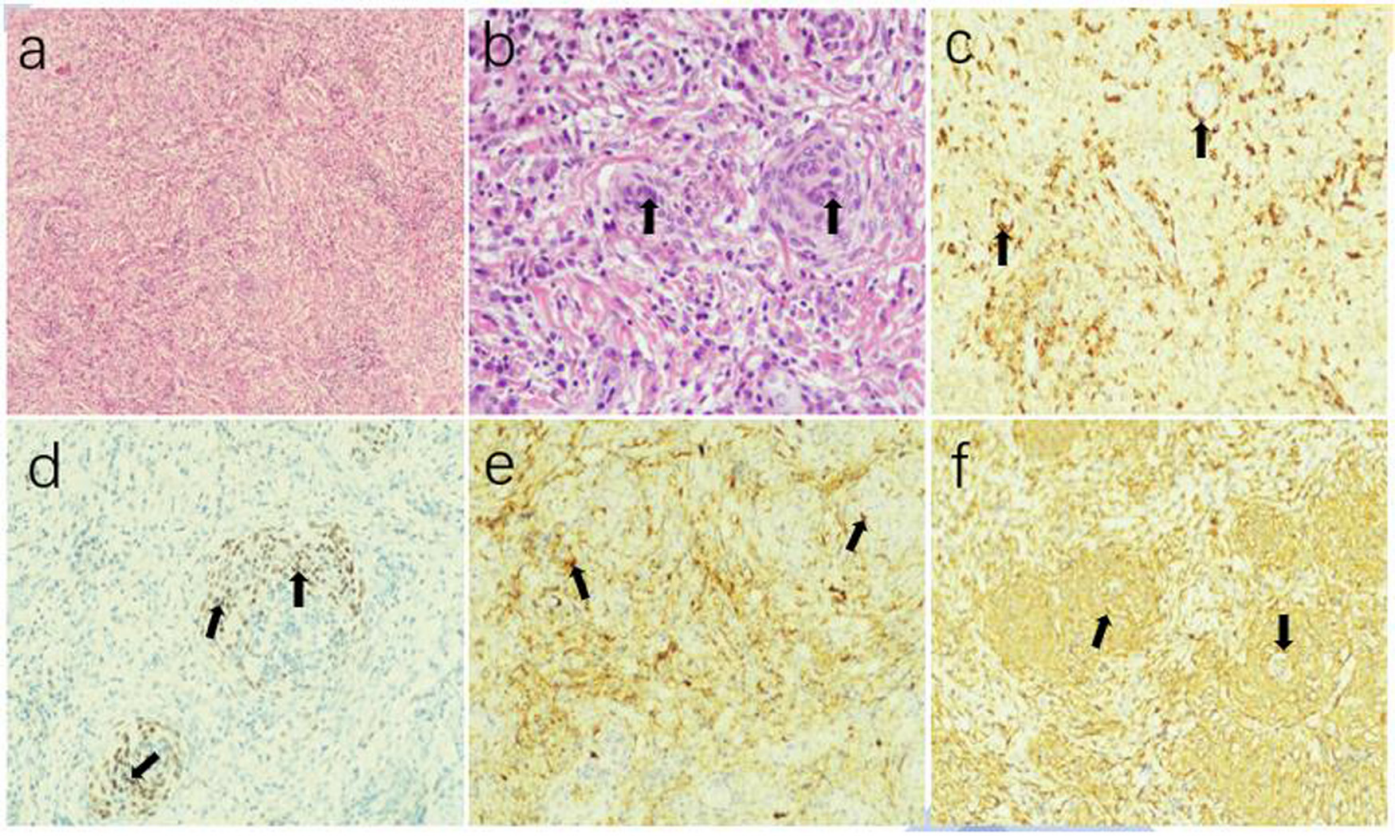
Photomicrographs demonstrating the histological features of the mass. Hematoxylin and eosin staining (magnification, ×100, ×400) (a and b) showing lymphoplasmacytes infiltrating the tumor stroma, overshadowing the meningioma component (arrows). Immunohistochemistry testing (c) showing epithelial membrane antigen positivity (arrows), (d) PR positivity (arrows), (e) S-100 positivity (arrows), and (f) vimentin positivity (arrows).

**Figure 3:**
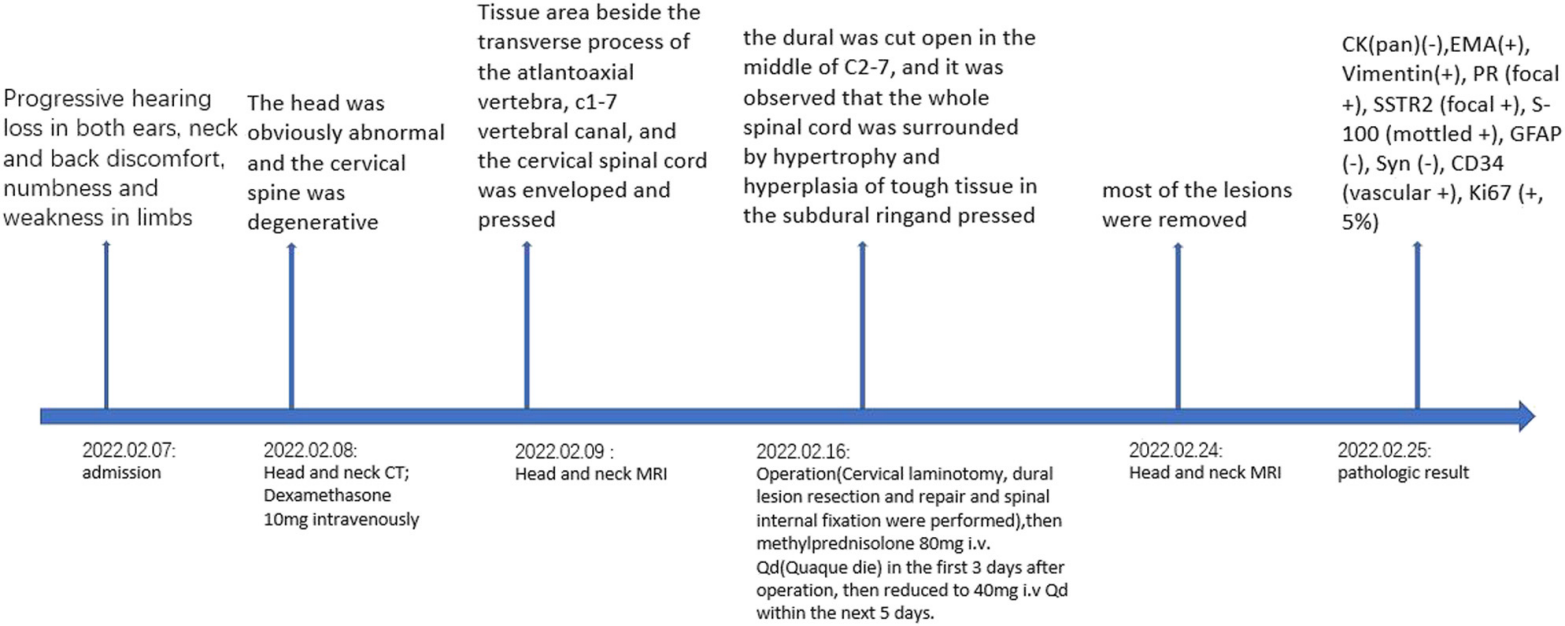
Timeline of the case presentation.


A 48-year-old Asian man was admitted to our hospital with progressive deafness in both the ears, neck and back discomfort, and limb weakness accompanied by numbness. The patient visited the hospital several times for bilateral hearing loss, neck swelling, and pain. The patient was diagnosed with hypertrophic dural inflammation, but treatment was ineffective. In the past 6 months, numbness and weakness in both the limbs were present, predominantly affecting the right side. These symptoms had significantly aggravated in the past month. Motor and sensory examinations revealed grade 4/5 right limb paralysis, grade 5/5 left limb paralysis, and normal muscle tension of the extremities. The bilateral pharyngeal reflex was weakened; the bilateral radial membrane reflex and knee reflex were strongly positive; Babinski’s sign, Kernig’s sign, and Brudzinski’s sign were negative; but the bilateral Hoffmann’s sign was positive. Other examinations were within normal limits. After admission, a computed tomography (CT) scan of the head and cervical spine revealed no obvious abnormalities (Figure 1a); however, magnetic resonance imaging (MRI) revealed multiple abnormal signals in the tentorium cerebelli, paradural area beside the saddle, soft tissue area beside the transverse process of the atlantoaxial vertebra, and the C1**–**7 vertebral canal, leaving the cervical spinal cord enveloped and pressed (Figure 1b and c). After multidisciplinary discussion, the patient was diagnosed as “hypertrophic dural disease? Malignant meningioma?” After completing the relevant examinations, cervical laminotomy, dural lesion resection and repair, and internal spinal fixation were performed. During the surgery, the dura was cut open in the middle of the C2**–**7 level, and the entire spinal cord was observed to be surrounded by hypertrophy and hyperplasia of the tough tissue in the subdural ring. However, because of the particular location and diffuseness of the lesions, only some of the cervical pulp lesions could be resected. Postoperative pathological examination was as follows (Figure 2): pathological visualization, C2**–**6 subdural space occupation: multiple grayish-yellow tissues with a total size of 4.53 cm × 0.5 cm; microscopically, C2**–**6 subdural space occupation combined with clinical and immunomarkers consistent with meningioma involvement (WHO grade I), with multiple inflammatory cell infiltration, considered to be lymphoplasmacytic. Immunohistochemical results were as follows: CK (pan) (−), EMA (+), Vimentin (+), PR (focal+), SSTR2 (focal+), S-100 (mottled+), GFAP (−), Syn (−), CD34 (vascular+), and Ki67 (+, 5%). After surgery, the patient’s muscle strength gradually improved, and reexamination with CT and MRI (Figure 1e and f) suggested that most of the lesions had been removed (Figure 3). The postoperative diagnosis was LPRM (WHO, I).

**Informed consent:** Informed consent has been obtained from all individuals included in this study.
**Ethical approval:** The research related to human use has been complied with all the relevant national regulations, institutional policies and in accordance with the tenets of the Helsinki Declaration, and has been approved by the authors’ institutional review board or equivalent committee.

## 3 Discussion

LPRM most often occurs in young and middle-aged adults, and the clinical features vary based on the site of the lesions. The symptoms are also nonspecific, often involving headache, nausea and vomiting, limb numbness, and hemiplegia. Infiltration of a large number of lymphocytes and plasma cells covering the basic components of meningioma tumors is common on histological examination [6]. Systemic hematological abnormalities, such as hyperglobulinemia and iron-refractory anemia, have been documented in more than 20% of the patients with LPRM [7]. In addition to the blood reports, a review of the relevant literature suggests that LPRM imaging is characterized by lesions with unclear boundaries and patchy shapes, obvious edema surrounding the lesions, and varying degrees of involvement of the adjacent brain tissues. Moreover, LPRM is more likely to have a diffuse growth than other types of meningiomas. For example, Han et al. [8] reported a case of diffuse LPRM in a 44-year-old man with lesions involving the sellar region, tentorium cerebelli, and skull base meninges from the clivus down to the sixth cervical spinal cord. Hirunwiwatkul et al. [9] also reported a case of LPRM extending from the sphenoid plateau down the slope to the foramen magnum. The mechanism of creeping growth observed in LPRM may be as follows: (1) the meningioma grows and erodes the meningeal lymphatic systems; (2) immune stimulation by tumor antigens causes meningiomas to express chemotactic agents, such as inflammatory and chemotactic cytokines, to assemble lymphocytes and plasmocytes; and (3) lymphocytes and plasmocytes change the microenvironment of the tumor and prevent gathering and adhering of meningioma components or inhibits the formation of peritumoral fibrin, thereby restricting the expanding growth of the tumor [10]. However, among the 120 LPRM cases reported in the literature, only ten (including this case) described diffuse growth LPRMs, accounting for approximately 8% of all LPRM cases. Comprehensive review of the existing cases can be found in Li et al. and is beyond the scope of this case report [10]. The proposed mechanism of the diffuse growth of LPRM was mainly based on the hypotheses proposed in previous cases, which requires more clinical data and experiments to verify.

Owing to the particularity of the diffuse growth of LPRM, this lesion is often difficult to diagnose using imaging before surgery. The differential diagnosis of LPRM with chronic inflammatory intracranial diseases is critical in its treatment. For example, idiopathic hypertrophic pachymeningitis, characterized by inflammatory fibrosis, can cause diffuse thickening of the dura mater, which is also known as an inflammatory pseudotumor [11]. This disease is mainly diagnosed based on exclusion. IgG4-related disease is a multisystem inflammatory disorder characterized by lymphoplasmacytoid infiltration, storiform fibrosis, and obliterative phlebitis [12], with or without elevated serum IgG4 levels. An IgG4/IgG plasma cell ratio of >40% is necessary for the diagnosis of IgG4-related disease [11]. Rosai–Dorfman disease is another inflammatory lesion of the dura that is usually negative for EMA expression. The diagnosis of LPRM is more likely if there is a significant positive expression of the EMA meningeal epithelium [13].

Currently, surgical resection is the best treatment option for LPRM. However, for LPRM with diffuse growth, the lesions are often extensive and difficult to completely resect through surgery. Furthermore, there are no unified standards for postoperative adjuvant therapy for LPRM. In a retrospective analysis by Tao et al. [5], radiotherapy was recommended for patients who underwent major or partial tumor resection. For patients with multiple tumor lesions, Wang et al. [14] reported a case of gamma knife radiotherapy for a local lesion with residual tumor progression 1 year after LPRM. The tumor in this case showed effective control, with dramatic tumor regression (3.14% per month). Therefore, radiotherapy is expected to be an effective treatment for LPRM. However, for LPRM with diffuse lesions, it is difficult to designate a radiotherapy target area. The lesions are located around the brainstem and cervical cord; thus, the radiotherapy target is difficult to define. Some authors regard the nature of LPRM as a mechanism of host immune resistance to the tumor, so it may bear some features of inflammation [15] and hormonal therapy has certain effect on LPRM. Corticosteroids could alleviate inflammatory response in tissues and suppress immune cell activity, thereby reducing the damage of inflammatory factors and immune cells to the central nervous system. In this case, the patient was also treated with dexamethasone 10 mg i.v. once before surgery, and methylprednisolone 80 mg i.v. q.d. in the first 3 days after surgery, which was reduced to 40 mg i.v. q.d. within the next 5 days. Hirunwiwatkul et al. [9] treated a diffuse growth LPRM with prednisone and azathioprine(60 mg/day prednisone and later 150 mg/day azathioprine.) for 6 months, followed by fractionated radiotherapy. Brain imaging examination showed the lesions slightly shrinked and there was no recurrence within 6 months. Zhang et al. [15] reported a case of LPRM mimicking pachymeningitis. The patient became lethargic after surgery. Intravenous methylprednisolone of 500 mg (q.d.) was administered for 3 days and then tapered off. Her mental status improved rapidly and dural enhancement on MRI fainted 2 weeks after corticosteroid treatment. Although some people believe corticosteroids are particularly effective in the adjuvant treatment, the dosage, pathways, and time of administration have not been clearly defined, which should be investigated further.

Currently, immunotherapy is administered to treat many types of tumors. The introduction of immune-checkpoint blockers and chimeric antigen receptor T cells has led to a paradigm shift in current oncology practices. The expression of immune checkpoints within the solid tumor microenvironment is an important factor influencing tumor-induced immunosuppression and evasion from the innate immune response in malignancies. Like many other tumors, meningiomas show upregulated expression of PD-L1 [16,17]; indeed, Han et al. [16] reported that PD-L1 is associated with poor survival outcomes, and may play a significant biological role in the aggressive phenotype of higher grade meningiomas. In the first report to focus on PD-L1 expression in LPRM, Gaggero et al. [18] reported the case of a 72-year-old man with right frontal LPRM, whose immunohistochemistry results showed high PD-L1 expression. As the applications of immunotherapy continue to progress, some scholars believe it is important to study the relationship between inflammation and tumoral lesions [18]. Overall, past research indicates that immunotherapy may improve the prognosis of patients, and is a promising therapeutic strategy based on the immunohistochemical characteristics of LPRM [8]. Although little is currently known about the immune landscape of meningiomas, robust evidence is still needed to confirm its clinical utility in the future. For our patient, radiotherapy and hormone therapy are expected to be applied in the later stages, and we will continue to observe their efficacy.

## 4 Conclusion

LPRM is a rare subtype of pathological meningioma. This disease mainly manifests in the young population, with no sex differences and no specific clinical symptoms. The diagnosis of LPRM is primarily based on pathological findings, and surgery is the primary treatment option. For LRPM with extensive lesions and diffuse growth, there is currently no effective treatment method owing to the rarity of this presentation. The risk of forced surgical resection is very high and may lead to permanent nerve injury or even death. Therefore, partial surgical resection with hormone therapy, radiotherapy, and immunotherapy are currently the most comprehensive treatment options available. However, the specific timing of immunotherapy, whether before or after surgery, or in conjunction with other treatment methods, as well as the specific efficacy of various treatment methods, requires more observation, summary, and in-depth mechanistic studies.
